# Bibliotherapy as a Non-pharmaceutical Intervention to Enhance Mental Health in Response to the COVID-19 Pandemic: A Mixed-Methods Systematic Review and Bioethical Meta-Analysis

**DOI:** 10.3389/fpubh.2021.629872

**Published:** 2021-03-15

**Authors:** Daniela Monroy-Fraustro, Isaac Maldonado-Castellanos, Mónica Aboites-Molina, Susana Rodríguez, Perla Sueiras, Nelly F. Altamirano-Bustamante, Adalberto de Hoyos-Bermea, Myriam M. Altamirano-Bustamante

**Affiliations:** ^1^Centro de Investigaciones Económicas, Administrativas y Sociales, Instituto Politécnico Nacional, Mexico, Mexico; ^2^Cross-Functional Group in Clinical Ethics, XXI Century National Medical Center, Mexican Social Security Institute, Mexico, Mexico; ^3^Facultad de Medicina, Universidad Nacional Autónoma de México, Mexico, Mexico; ^4^Departamento de Traducción y Ciencias del lenguaje, Pompeu Fabra University, Barcelona, Spain; ^5^Servicio de Endocrinología, National Institute of Pediatrics, Mexico, Mexico; ^6^Metabolic Diseases Research Unit, XXI Century National Medical Center, Mexican Social Security Institute, Mexico, Mexico

**Keywords:** bibliotherapy, litherapy, mental health, coronavirus disease 2019, pandemic, values, bioethics, systematic review

## Abstract

**Background:** A non-pharmaceutical treatment offered as psychological support is bibliotherapy, which can be described as the process of reading, reflecting, and discussing literature to further a cognitive shift. The coronavirus disease 2019 (COVID-19) pandemic demands a response to prevent a peak in the prevalence of mental health problems and to avoid the collapse of mental health services, which are scarce and inaccessible due to the pandemic. Thus, this study aimed to review articles on the effectiveness of bibliotherapy on different mental health problems.

**Methods:** A systematic review was conducted to examine relevant studies that assess the effectiveness of bibliotherapy in different clinical settings as a treatment capable of enhancing a sense of purpose and its surrounding values. To achieve this, a systematic review, including a bioethical meta-analysis, was performed. A variant of the PICO (Participants, Intervention, Comparison, and Outcome) model was used for the search strategy, and the systematic review was conducted in three databases: PubMed, Bireme, and OVID. Inclusion criteria were relevant studies that included the keywords, excluding documents with irrelevant topics, studies on subjects 15 years or younger, and in languages besides Spanish or English. Starting with 707 studies, after three rounds of different quality criteria, 13 articles were selected for analysis, including a hermeneutic analysis, which was followed by a fourth and final recovery round assessing bibliotherapy articles concerning healthcare workers.

**Results:** Our findings showed that through bibliotherapy, patients developed several capacities, including the re-signification of their own activities through a new outlook of their moral horizon. There are no research road maps serving as guides to conduct research on the use of bibliotherapy to enhance mental health. Additionally, values such as autonomy and justice were closely linked with positive results in bibliotherapy. This implies that bibliotherapy has the potential to have a positive impact in different settings.

**Conclusions:** Our contribution is to offer a road map that presents state-of-the-art bibliotherapy research, which will assist institutions and healthcare professionals to plan clinical and specific interventions with positive outcomes.

## Introduction

Amid the coronavirus disease 2019 (COVID-19) pandemic, a pressing problem faced by different health ministries is the mental health of the population, this includes both those who have been social distancing and staying indoors for long periods at a time and those considered essential workers who have continued laboring despite the considerable risk—among them are healthcare workers (HCWs). This has exposed the population to a variety of psychological diseases such as sleep disorders, depression, anxiety, and burnout. These disorders affect a broad spectrum of individuals from different backgrounds and across ages (1–3). If not addressed, the prevalence and financial burden of mental health disorders in communities affected by COVID-19 will grow exponentially. Furthermore, both the healthcare and socioeconomic systems will collapse if a significant improvement is not made in the diagnostic approach, prevention, and non-pharmacological treatment of psychological disorders.

Mental health problems can be addressed through a plethora of available treatments, such as psychosocial therapies or cognitive behavioral therapy (CBT), which are provided by trained psychologists (4). However, regardless of the availability of different treatments, few people with mental health problems have sought help during the COVID-19 pandemic (5). This setting, along with the need for social distancing, poses a challenge when considering available treatments for improving mental health.

One of the available non-pharmacological treatments in psychological literature is bibliotherapy (6). This is defined as reading as a guide to therapeutic change; bibliotherapy has been studied by mental health scientists in recent years as a tool, different from traditional interventions, that improves the readers' lives (7, 8). Though several definitions have been created to conceptualize *bibliotherapy*, they all focus on three essential elements: reading material for inside- or outside-session use, a therapeutic and achievable goal, and personal improvement.

Bibliotherapy is better understood as the process of reading, reflecting upon, and discussing literature (personal narratives and stories). This discussion of curated literature promotes cognitive shifts within the reader (9). It is crucial to note that bibliotherapy differs from self-help strategies as the reflection and discussions of literature take place in a structured setting (10). The reading material is also subjected to scrutiny and has a specific purpose or problem that it addresses.

The use of books in a systematic clinical setting offers the possibility of improving mental health at a low cost. In addition, it represents an alternative for those who are hesitant to receive treatment for mental health problems (11). For essential workers, including healthcare professionals, it is essential to be treated and to get help. Not only do mental health problems lead to moral distress, but they can be associated with loss of values when treating patients, which in turn reduces the quality of care.

Bibliotherapy has shown positive results for various mental disorders in different trials, which justify the rational and empirical evaluation of this approach. However, in the existing literature, it remains to be studied whether bibliotherapy can enhance values that contribute to obtaining a sense of purpose. Therefore, the main objective of the present work is to review the principal studies that assess the effectiveness of bibliotherapy as a treatment to enhance a sense of purpose and values in those with different mental health problems. Based on these results, healthcare professionals and institutions can plan clinical and specific interventions that are well-tested, assessed, and valued and show clinical effectiveness in improving mental health and the work environment. As a result, different mental health issues, such as anxiety, depression, sleep disorders, and burnout, can be addressed through bibliotherapy.

## Methods

A diagnostic screening was performed on five databases: PubMed, Bireme, OVID, Philosopher's Index, and JSTOR, searching for the most recent articles on the use of bibliotherapy as a non-pharmacological intervention to help mental patients and HCWs. Subsequently, a systematic review was conducted up to February 2018 to obtain original articles about available literature-based non-pharmacological treatments (bibliotherapy). This search was complemented with a screening up to 2020, incorporating the COVID-19 pandemic. The search strategy was based on the PICO (Participants, Intervention, Comparison, and Outcome) approach coupled with the PRISMA (Preferred Reporting Items for Systemic Reviews and Meta-Analyses) checklist (12). However, the comparison variable was removed due to the research being focused only on bibliotherapy as an intervention. The PIO (Participants, Intervention, and Outcome) strategy, which includes participants or problems, the intervention or exposure, and outcomes, was used to systematically search all databases. This type of modified search derived from the PICO model has also been used in other systematic reviews (13–15).

### Search Strategy/Literature Search

The search strategy was carried out on five computerized databases: PubMed, Bireme, Philosopher's Index, JSTOR, and OVID. The first database used was PubMed, produced by the National Library of Medicine (a public body that depends on the National Institutes of Health of the United States), which, according to Cochrane (16), contains approximately 16 million references to journal articles from the year 1950 onward, as well as 5,200 indexed journals. This makes PubMed/Medline the most widely used database in the health sciences field globally. Subsequently, a search was carried out in the Virtual Health Library (VHL) database, produced by Bireme (Latin American and Caribbean Center for Information in Health Sciences), which is a specialized center of the Pan-American Health Organization (PAHO). There is a considerable importance to this database because it contains (indexed) the most relevant scientific literature journals from Latin America and the Caribbean. We performed the search as well in Philosopher's Index, which is a premier database designed to find publications of interest in the field of philosophy. The axiological analysis is the great interest for philosophers worldwide. Following is JSTOR that is a cross-functional database.

Finally, a search was carried out in OVID, the world's most trusted medical research platform, which has been a vital part of healthcare for over 20 years. OVID's flagship platform is the leading choice, globally, among clinicians, researchers, educators, and students in the medical, scientific, and academic fields.

**Figure 1:**
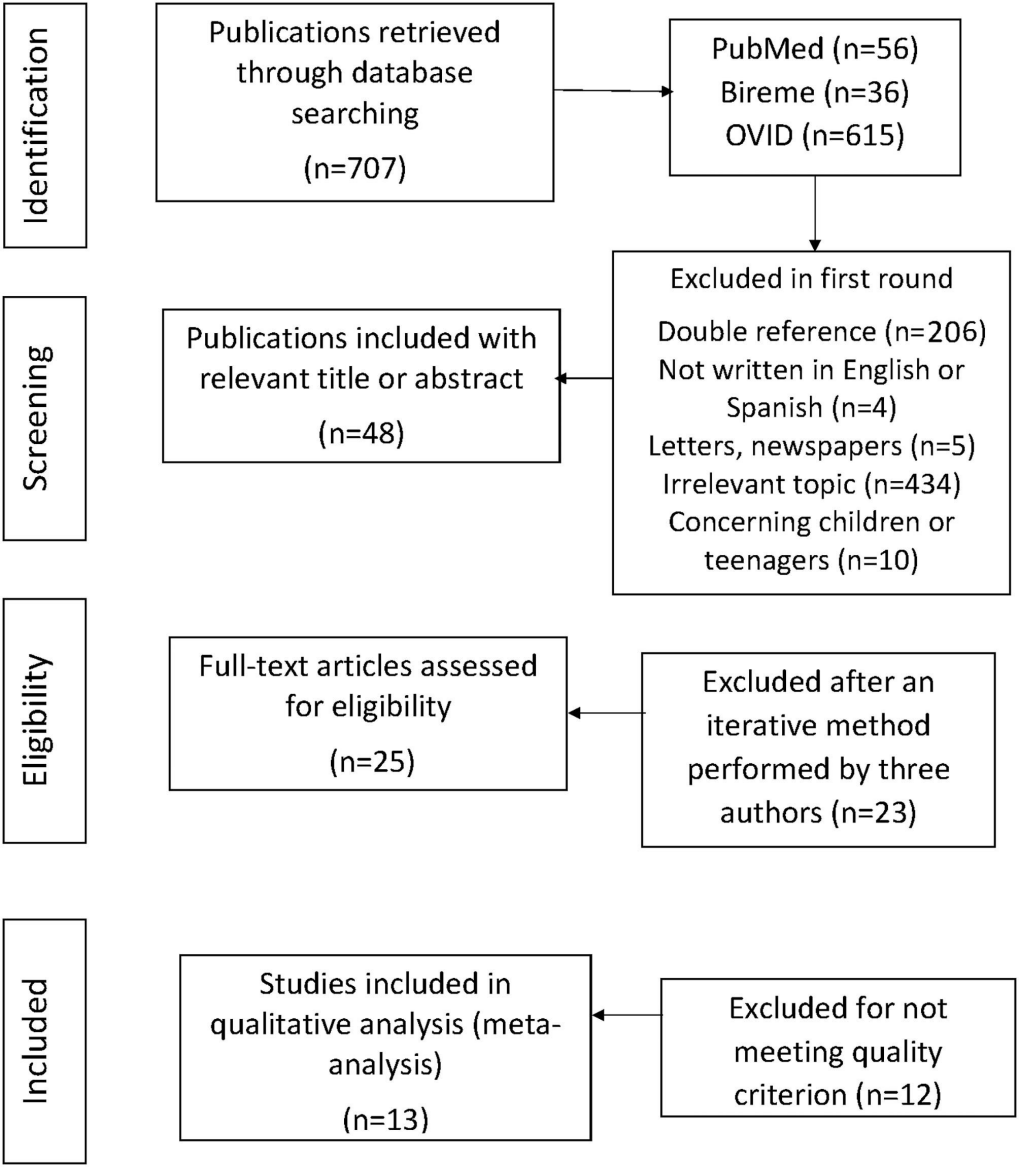
Flowchart. It shows the selection process used to retrieve the final 13 articles. In the first selection round, 707 articles were obtained from three computerized databases (PubMed, Bireme, and OVID). Works with double references, irrelevant topic, or not written in English or Spanish were excluded during the second round of screening, leaving 25 articles. The third round of selection included a quality criterion in which the full text was read. Finally, 13 studies with over 80% were selected for a hermeneutic analysis.

Mesh terms were used to review this phenomenon. The following keywords were used for Participants: “healthcare personnel,” “healthcare professional,” “healthcare manpower,” “physician,” “doctor,” “nurse,” “social worker”; Intervention: “bibliotherapy”; and Outcome: “liberty,” “empowerment,” “tolerance,” “justice,” “benevolence,” “equity,” “respect,” “charity,” “beauty,” “autonomy,” “purity,” “ethical values,” “axiology,” “personal identity,” and “dignity.” The Boolean operator “AND” was used to link PIO variables, while the operator “OR” was used to combine keywords from the same variables. This search strategy was used to obtain relevant articles from each database, as shown in Figure 1. All references were stored in Mendeley Desktop. Due to the scarce results on health workers, a redirection to the general population was done using the screened papers that were already in our curated database.

### Eligibility Criteria

In the first round, articles were selected from every electronic database and were screened for relevance based on their title and abstract. Works with a double reference, on participants 15 years old or younger, irrelevant topic, related to letters or books, and not written in English or Spanish were excluded. Before the second round, three authors performed an iterative review to confirm the actual relevance of each paper.

In the second round of selection, the full text of the articles was considered. A quality criterion, as seen in Supplementary Table 1, was used to assess the methodological value of each reference. The articles were read and classified according to the following criteria: (a) clear investigation objectives, (b) inclusion of a research question according to the objectives, (c) adequate and solid methodology, (d) terminology definition, and (e) results according to the objectives. Each criterion was worth 20%, with a maximum quality score of 100%.

In the third round, 13 articles met the optimal quality criteria (higher than 80%). Each article was analyzed and codified using Atlas. ti software to highlight the backgrounds of the bibliotherapy interventions, methodological elements, and results. All articles were codified to develop a deeper qualitative analysis based on the different codes, their relations, co-occurrences, and their networks. The bioethical meta-analysis searched for networks that could link bibliotherapy as an intervention to enhance social, ethical, and professional values.

Finally, a fourth round was executed as a recovery round to retrieve any articles directly linked to the intervention of bibliotherapy for HCWs.

## Results

### Relevance Assessment of the State of the Art of Bibliotherapy

The first round yielded 707 initial articles, after which 25 articles were retrieved in the second round of selection; lastly, 13 articles met the quality criteria and were retained for further analysis (Figure 1, Supplementary Figure 1).

Studies that had clear research questions and objectives, definitions of the measured concept, valid measuring instruments, detailed description of the methods, information of the targeted population, characteristics of the participants, addressed missing values, and used appropriate statistical analysis were considered. The results are shown in Table 1.

**Table 1:** Quality criteria for the second round of selection.

**First author, year of publication**	**Setting**	**Sample size**	**Sample characteristics**	**Design study**	**Study quality**
Bilich (2008)	Psychotherapy	84 participants	Ages between 18 and 60 scored between 10 and 29 on BDI	Control vs. Experimental 1 vs. experimental 2	87.5%
Bokey (2002)	Literary works analysis	–	–	–	–
Buwalda (2009)	Pilot study	40 participants	Mean age 43, with hypochondriacal complaints.	Treatment condition vs. waiting list condition	100%
Chien (2016)	Clinical trial	116 caregivers	Chinese people, mean age 50 years, two thirds were females	Only experimental group	100%
Cohen (1992)	Literature for woman analysis	–	–	–	–
Cohen (1993)	Books analysis	–	–	–	–
Cohen (1994)	Interview	8 persons	Participants between 26 and 54 years	Participants interviewed or 90 min	50%
Evans (1999)	Clinical trial	34 patients	Aged between 16 and 50 with deliberated self-harm episode	Experimental vs. treatment as usual	100%
Harrison (2001)	Literature analysis	–	–	–	–
Hodgins (2007)	Booklets delivery mail setting	169 participants	Over 17 years and meet criteria for pathological gambling	Experimental vs. experimental	87.5%
Joling (2011)	Psychological intervention	119 elderly eligible people	Elderly people aged 75 years and older	Usual treatment plus cognitive behavioral bibliotherapy intervention vs. treatment as usual	100%
Kaldo (2012)	Psychotherapy	89 persons	Participants mainly Caucasian, well educated, mean age 49.1	Control vs. experimental	87.5%
Kohutek (1983)	Correlational setting	42 people	Incarcerated males, average age 34	Experimental groups only	87.5%
Lanza (1991)	Literature review	–	–	–	–
Macdonald (2013)	Bibliotherapy *via* local services	157 participants	Exclusion criteria: under 16, without reasonable English literacy, diagnosed with mental illness	Experimental group	87.5%
McAllister (2014)	Memoirs works analysis	–	–	–	–
McArdle (2001)	Theoretical analysis	–	–	–	–
McKenna (2010)	Interviews	11 volunteers	Mean age 41 years, age range 27–64 years 5 men, 6 women	Interviews	62.5%
Naylor (2010)	Medicine center	38 persons	From 22 to 83 years old. Predominantly female and Caucasian.	Treatment as usual vs. experimental	50%
Porter (2008)	Mailed surveys	21 persons	Not specified	Mixed-methods design	50%
Reeves (2005)	Clinical trial	19 patients	18 years old, experiencing mild to moderate stress/anxiety	Only experimental group	50%
Reeves (2010)	Control trial	43 clients	Age limit 18 upward and primary presenting problem	Treatment group vs. control group	100%
Van Lankveld (1999)	Couple's therapy	246 couples	Older than 16 with heterosexual relationship and a sexual disorder	Voluntary response sample and waiting list group	87.5%
Volpe (2015)	Hospitalized patients	41 patients	Patients had a diagnosis of schizophrenia, schizoaffective and bipolar disorder	Control group vs. experimental group	100%
Wright (2000)	Participants who completed a previous study	45 patients	Patients with panic attacks	Wait list group control vs. experimental	87.5%

*BDI, Beck Depression Inventory-Revised*.

These 13 studies were analyzed in depth to highlight the strengths of each work. The following considerations were added to the analysis: if it was treatment or prevention, targeted disorder, duration of intervention, sessions per week, instrument used, and main results (Table 2).

**Table 2:** Description of the articles in the third round of selection.

**First author, year of publication**	**Sample**	**Design study**	**Treatment or prevention**	**Disorder**	**Duration**	**Sessions per week**	**Books and booklets used**	**Test**	**Results**	**Impact of intervention**
Bilich (2008)	84 participants	Control vs. Experimental 1 vs. experimental 2	T	Depression	20 weeks	30 min telephone contacts	The Good Mood Project (2003)	BDI II DASS 21 K 10	Bibliotherapy as a potential self-help resource	
Buwalda (2009)	40 participants	Treatment condition vs waiting list condition	T	Hypochondriasis	Not specified	Not specified	Doctor, I hope it's nothing serious?	The Groningen illness attitude scale Spielberg state trait anxiety questionnaire Beck Depression Inventory	Bibliotherapy is an efficient aid in reducing hypochondriacal complaints	
Chien (2016)	116 caregivers	Only experimental group	T	Family caregivers	22 months	20-week modules, 1 h per session	Clinician-supported problem-solving bibliotherapy	Family burden interview schedule Experience of caregiving inventory	Bibliotherapy produces moderate long-term benefits to caregivers and first episode psychosis patients	
Evans (1999)	34 patients	Experimental vs. treatment as usual	T	Deliberate recurrent self-harm	6 months	Not specified	Manual assisted cognitive behavior therapy	Social Functioning Questionnaire Parasuicide History Interview	Feasibility of MACT as a therapeutic procedure for deliberate self-harm	
Hodgins (2007)	169 participants	Experimental vs. experimental	T	Pathological gambling	6, 24, 52 weeks	Not specified	Series of relapse prevention booklets	Gambling severity GASS CES-D	Extended relapse prevention bibliotherapy to problem gamblers does not improve outcome	
Joling (2011)	119 elderly eligible people	Usual treatment plus cognitive behavioral bibliotherapy intervention vs. treatment as usual	T	Subthreshold Depression	12 weeks	1 per week	Coping with depression self-help manual	CES-D	Significant change statistically and clinically relevant but not better than TAU	Bibliotherapy is only effective if patients are motivated and acknowledge their depression
Kaldo (2012)	89 persons	Control vs. experimental	T	Insomnia	6 weeks	Telephone intervention 15 min	Not specified	Insomnia Severity Index		
Kohutek (1983)	42 participants	Experimental groups only	T	Incarcerated male inmates	4 weeks	Not specified	Three settings of readings: personal growth packet, rational-growth packet, and general readings packets.	Levenson Locus of Control	Bibliotherapy may facilitate self-concept and internal locus of control as well as usual treatment. No differences between treatments	
Macdonald (2013)	157 participants	Experimental group	T	Mild mental health that does not require referral to psychological or psychiatric services	Not specified	Not specified	“Read yourself well” scheme	Clinical outcomes in routine evaluation questionnaire General health questionnaire	Positive use of self-help written materials in the management of minor mental health problems	
Reeves (2010)	43 clients	Treatment group vs. control group	T	Mild to moderate stress and anxiety	5 months	30–40 min per session	Assisted therapy	The hospital anxiety and depression scale The clinical outcomes in routine evaluation score Satisfaction questionnaire Compliance questionnaire	Assisted self-help package produces clinically significant improvements above of those in waiting list	
Van Lankveld (1999)	246 couples	Voluntary response sample and waiting list group	T	Sexual dysfunctions	Not mentioned	Not mentioned	Not specified	CIDI GRISS SCL90 IBCS MMQ IPSO	No differences between bibliotherapy group and control group	
Volpe (2015)	41 patients	Control group vs. experimental group	T	Functional psychosis	6 months	90 min	Serious literature materials	Brief Psychiatric rating scale The Personal Health Questionnaire Depression Scale Mini-Mental State Examination	Statistical significant improvement of cognitive and psychosocial functioning	
Wright (2000)	45 patients	Wait list group control vs. experimental	T	Panic Attacks	6 months	Not specified	“Coping with panic” Manual	Panic Attacks Symptoms and Cognitions Questionnaires, Avoidance Questionnaire, Coping Strategies (CS) and Confidence in CS Questionnaires, Beck Depressive Inventory	Significant reductions of frequency of panic attacks, panic cognitions, anticipatory anxiety, avoidance and depression.	

*BDI II, Beck Depression Inventory-Second Edition; CES-D, Center for Epidemiological Studies - Depression Scale; CIDI, Composite International Diagnostic Interview; DASS, Depression, Anxiety and Stress Scale - 21; GASS, Gambling Abstinence Self-efficacy Scale; GRISS, Golombok Rust Inventory of Sexual Satisfaction; IBCS, Intimate Bodily Contact Scales; IPSO, Interactional Problem Solving Questionnaire; K10, Kessler Psychological Distress Scale; MMQ, Maudsley Marital Questionnaire; SCL90, Symptom Checklist-90*.

One of the main findings showed that all studies addressed a treatment perspective rather than a prevention perspective. Three of these studies did not specify the length of the intervention, and two out of these 13 studies did not mention the kind of literature they used (17, 18).

Considering the main results of these studies, three of them found no differences between the bibliotherapy group and control group (17, 19, 20). However, results from four other studies indicated that bibliotherapy may facilitate self-concept and an internal locus of control (20–23).

Another study showed that with bibliotherapy, though there was a significant change, it did not result in a better intervention than traditional treatment (19). Another study pointed out that bibliotherapy was better than being on the waiting list (24). Finally, the remaining three studies found that bibliotherapy was a potential self-help resource (10, 24, 25).

Of the 13 studies included, two used bibliotherapy as an additional treatment (19, 23), while the remaining 11 studies tested bibliotherapy as the main treatment. One paper described pretreatment evaluation (17), while 12 studies evaluated pretreatment and posttreatment. None of the studies showed any adverse effects of using bibliotherapy as a treatment; however, two studies described the effect as not adverse, although this finding was not significant (19, 26).

Regarding improvement, out of the 13 studies, six did not specify the percentage of patients who improved (11, 20–22, 24, 27). Bilich et al. (10) describes that 31% of the total sample showed clinically significant changes (10), Hodgings et al. (26) reported a 23% improvement, and Kaldo et al. (18) reported that 68% of the progress was made in the bibliotherapy group (26). Furthermore, Macdonald et al. (25) mentioned a 100% advance in participants receiving bibliotherapy, and Wright et al. (23) specified an 89% recovery of the participants in the experimental manipulation condition.

The three studies found in the recovery round, which assessed articles directly linked with bibliotherapy in HCWs, showed the impact that different literary works can have. While two studies focused on the nursing population, Amar (28) examined the impact of personal stories on nursing students' education and Harrison (29) studied the use of imaginative literature in scholarly inquiry, Andersonet al. (30) assessed the importance of empathy in both physicians and patients through the use of graphic stories. Taken together, all three articles highlight the positive impact of bibliotherapy for healthcare personnel.

### Semantic Networks as an Initial Compass

**Figure 2:**
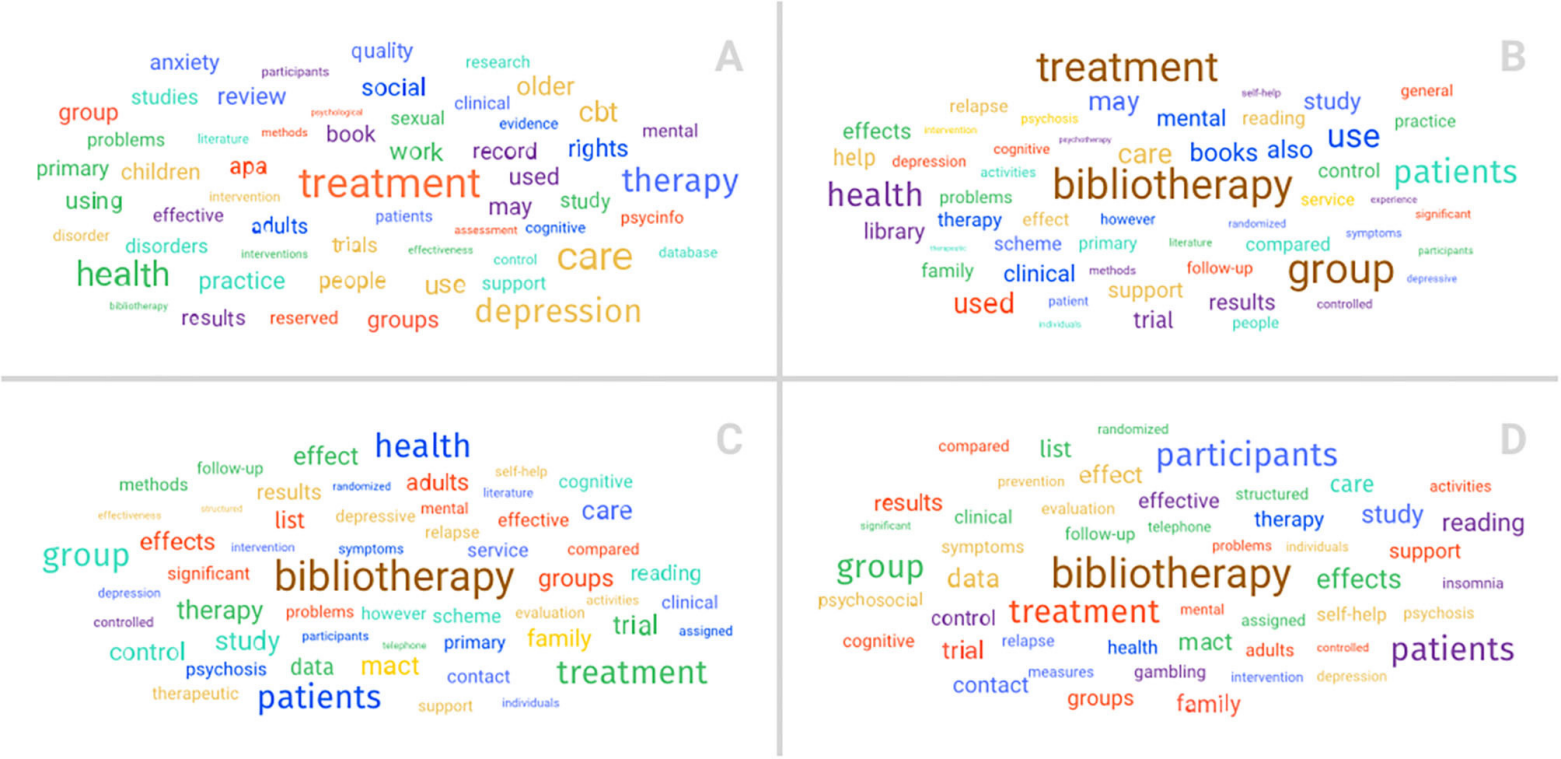
Keyword frequency. Analysis of database stored in Mendeley regarding the words in the title, keywords, and abstract. **(A)** Four hundred eighty-eight articles were retrieved in the first round, **(B)** 35 in the second, **(C)** 25 in the third, and **(D)** 13 in the fourth. Results showed a coincidence in the most frequent words.

The database stored in Mendeley was analyzed for the frequency of the terms used in the title, keywords, and abstract of each work according to the search parameters specified in the Methods section. The results of the first search yielded 488 articles (without double references) (Figure 2A), and five studies were discarded because no abstract was found. Based on this volume of articles, the word count was modified to consider only terms with 75 coincidences, meaning those with a significant frequency (Figure 2).

The five most common terms in the literature records were treatment (560), care (386), health (378), depression (316), and interventions (271). At this point in the research, bibliotherapy did not appear as a frequent word, since there were only 126 coincidences, placing it at the 38th position. As bibliotherapy was the key concept of the present study, another round was conducted to obtain relevant studies for the analysis.

In the second round, which yielded 35 documents, three were discarded due to the lack of an abstract. The most frequent terms in those articles were bibliotherapy (62), treatment (40), group (35), patients (31), and health (28), where the terms treatment and health coincided with the previous selection (Figure 2B).

In the crossed iteration of the second round, 25 articles were retrieved, where two were again discarded because they did not have an abstract. The same list of terms, as in the previous round, was found; nevertheless, based on the significantly reduced volume of articles, the coincidences were not limited. The most frequent words in these documents were bibliotherapy (40), treatment (30), group (24), health (22), and patients (22) (Figure 2C).

These new results showed a coincidence in the most frequent words except in the order of health and patients, since these terms were inverted in the present third round. It is worth noting that bibliotherapy was the most frequent word in the 25 articles that point out the proper delimitation of each selection round.

In the third round of selection, 13 final articles were retrieved. One of them did not have an abstract. The same list of terms as in the previous search was found, and there was no restriction on the number of elements in the word count. The most frequent words were bibliotherapy (23), group (23), treatment (21), patients (17), and participants (15) (Figure 2D).

There was a coincidence in four out of the five terms compared to the previous round of selection. This change shows that the articles had a strong methodological component in the implementation of bibliotherapy, which is consistent with the present systematic review.

In the last round, we also researched the complete text of the 13 articles; the most frequent words were group (427), treatment (402), bibliotherapy (295), participants (288), study (258), depression (244), health (191), mental (160), patients (160), and clinical (156).

### Findings From Bibliotherapy and Values

Hereafter, a table was created to show values that were considered in each paper (Supplementary Tables 1, 2). The different values that a study impacted are shown, with a cognitive shift on the part of the participants or considered relevant, on how they view their own life, their treatment, or everyday activities. By doing this, it was found that all articles spoke about gaining autonomy from bibliotherapy, 10 works addressed liberty as a central value in the intervention, and five articles regarded being proactive toward treatment. No articles that highlighted honesty, veracity, justice, or beauty were found (as shown in Table 3). To specify what is understood for each value, examples of quotes can be seen in Table 4.

**Table 3:** Values considered in the last round of review.

**References**	**Fidelity**	**Autonomy**	**Liberty**	**Equality**	**Respect**	**Tolerance**	**Interest**	**Love**	**Service attitude**	**Charity**	**Purity**
Bilich et al. (10)		X	X				X				
Buwalda and Bouman (27)	X	X	X								
Chien et al. (11)	X	X	X		X	X		X	X	X	
Evans et al. (21)		X	X					X			
Hodgins et al. (26)		X	X		X		X	X			X
Joling et al. (11)		X	X		X		X				
Kaldo et al. (18)		X	X								
Kohutek (20)		X	X								
Macdonald et al. (25)		X									
Reeves (24)		X								X	
van Lankveld et al. (17)	X	X		X	X		X	X			
Volpe et al. (22)		X	X				X				
Wright et al. (23)				X	X						

**Table 4:** Instances of values.

**Value**	**Example**
Fidelity	The participants valued the book substantially, by rating it and its separate chapters as highly useful (27).
Autonomy	Our study demonstrated that structured group reading activities may exert some positive influence on clinical symptomatology and cognitive and psychosocial functioning in patients with psychosis (22)
Liberty	The main prison population may be less defensive about the topic in that they have more freedom and, therefore, can benefit more from the principles of rational growth (20)
Equality	Our findings strongly support the theoretical notions of the existence of distinct types of sexual difficulty, associated with different personal and relational characteristics (17).
Respect	Individual empowerment, which is one of the aims of bibliotherapy, contributes to the development of problem-solving skills and increases self-esteem and self-efficacy (19).
Tolerance	The CSPSB also experienced a greater reduction in negative appraisals of caregiving and more positive experiences of family relationships/communication with their FEP relative than the UOFS group (11).
Interest	The findings are also supported by several researchers who emphasize the importance of individuals who are engaged in “self-help” treatment having telephone contact with mental health workers, particularly in rural settings (10).
Love	The results nonetheless suggested that the intervention might be effective in reducing the number and frequency of self-harm episodes, with simultaneous reduction in depressive symptoms (21).
Service attitude	The 5-month CSPSB demonstrated very positive effects on family caregiving at 12-month follow-up (22).
Charity	Contact with the clinician was repeatedly identified as being valued and is consistent with the previous studies (24).
Purity	These participants appeared “ready to change” and were motivated enough to initiate some self-change as a result of being involved in a research study. The extended contact (i.e., follow-up interviews) have also contributed to the maintenance of their motivation (26).

*CSPSB, Clinician-Supported Problem-Solving Bibliotherapy; FEP, First-Episode Psychosis; UOFS, Usual Outpatient Family Support*.

**Figure 3:**
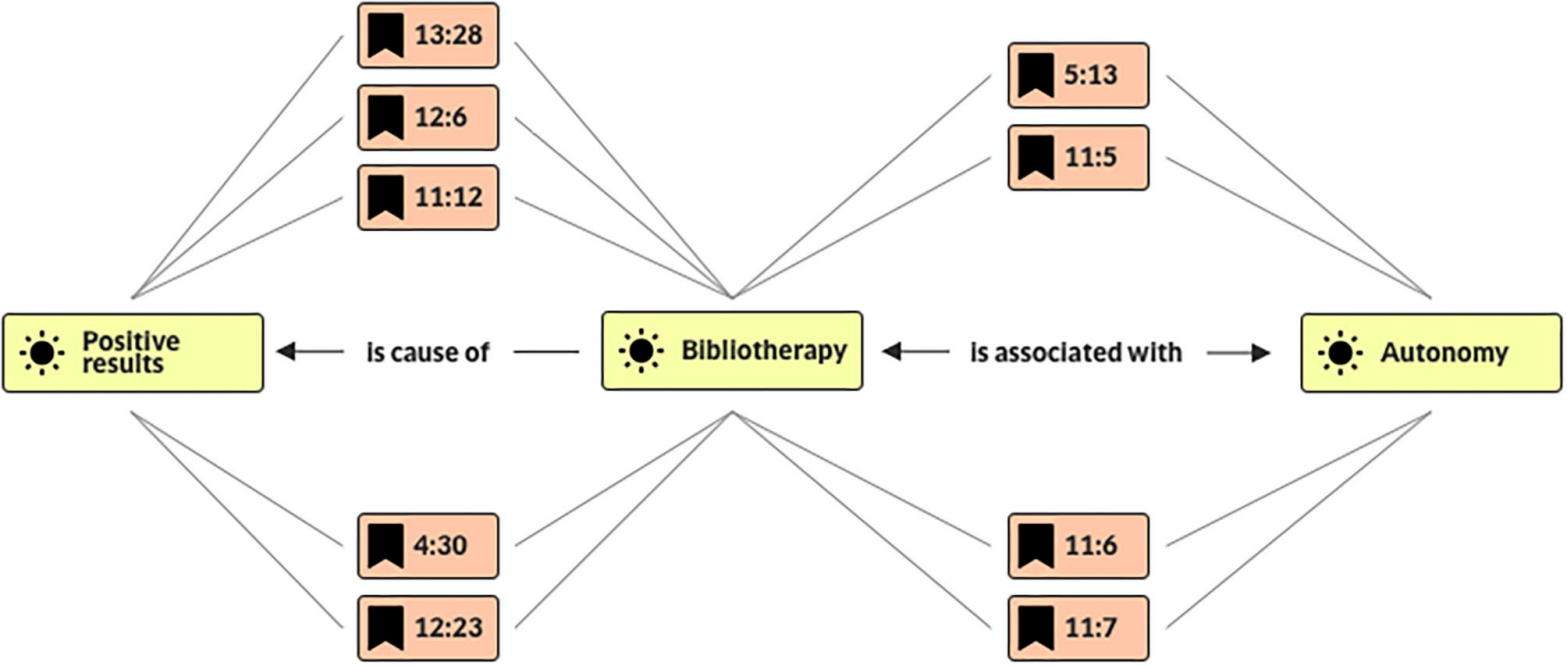
Values linked to bibliotherapy. Network of values performed in Atlas. ti. It illustrates how values such as autonomy, liberty, and justice are associated with positive results regarding bibliotherapy treatment. The figure depicts how autonomy is a crucial value found in all 13 texts of the final round as well as a network between values. The identification numbers refer to each of the 13 texts and the line in that text.

To develop the hermeneutic analysis, each paper was read in depth and codified using Atlas. ti software to create networks of terms. One example is shown in the next image (Figure 3), where values such as liberty, autonomy, and justice are closely linked and associated with positive results in bibliotherapy.

In the third round of selection, the analysis showed that autonomy, justice, and freedom were the values most frequently discussed in these papers. Autonomy was a value closely linked with positive results in the case of bibliotherapy because this is one of the ways to promote a self-help treatment that could promote empowerment and allow for the control of an increasing number of situations in patients' lives, enable them to solve their own problems, and acquire the skills necessary to do it (19). These studies showed improvements related to self-concept and locus of control, which also reflect improvements in the autonomy of a patient. However, bibliotherapy was also found to promote autonomy in the participation of a patient in their own treatment by being proactive when given access and benefiting from the information about their illness or condition and lowering the caregiver's burden by improving compliance and reducing anxiety episodes (27). Values such as autonomy and proactiveness also showed an impact on values, such as liberty, due to the enhanced self-efficacy and self-concept, an improvement in life possibilities, options open to patients with fewer symptoms, and better control of their day-to-day lives.

Meanwhile, justice may also be considered a value linked to bibliotherapy in the view of these works because it allows for a great deal of access to this form of intervention. Wright et al. (23) states that bibliotherapy is accessible to individuals who may be geographically or otherwise isolated; it is also a valuable form of treatment for those with limited economic resources and helps caregiving institutions to pay attention to larger groups with limited personnel. Bibliotherapy also allows for a more private way to address these health issues, without having to deal with negative perceptions or the reticence of those not willing to share their concerns with others.

These considerations may also be applied to a specific population, that is, HCWs who, due to the COVID-19 pandemic, have been under significant stress and their mental health has been threatened due to the conditions they face, moral burdens, and workload. In the literature reporting the specific case of mental health among HCWs, we found that all the previous stress factors signaled to widespread anxiety- and depression-related disorders (31–33). There was no report on bibliotherapy used to help HCWs; however, it stands as a viable option due to the logistic difficulties of offering standard treatments amid the pandemic.

### A Hermeneutic Analysis on How Bibliotherapy Works

The analysis of these texts stemmed from phenomenological and hermeneutic approaches. The latter's, insofar as it concerns a naive reading, results are shown in Table 5.

**Table 5:** Hermeneutic analysis.

**Source**	**Methods analysis**	**Results**	**Discussion**
Characteristics of couples applying for bibliotherapy *via* different recruitment strategies: A multivariate comparison Jacques J. D. M van Lankveld, 2008	They took 140 couples who responded to a call, and 106 couples who attended a clinic. The couples were interviewed separately	The distribution of sexual dysfunction varies between men and women. Diagnoses of varied disorders in men and women.	The objective was to know the sexual dysfunctions of couples who actively sought bibliotherapy. No multivariate differences were found between the two groups: Media Group and Sexology Group.
Involving Clients in Treatment Methods: A Neglected Interaction in the Therapeutic Relationship Viktor Kaldo, 2015	89 participants were taken who fulfilled the criterion of insomnia, predominantly educated white women.	The result was that carrying out a treatment with the therapist's help significantly increases the benefits of such treatment.	Therapeutic support is more effective with the involvement of the patient, so it is concluded that the relationship and methods depend on said interaction.
Effectiveness of Bibliotherapy Self-Help for Depression with Varying Levels of Telephone Helpline Support Linda L. Bilich, 2008	The effectiveness of telephone support was examined in 84 subjects with mild to moderate depression.	Clinically significant improvements in two of the control groups.	Participation was not spontaneous as the participants were contacted *via* advertisements. The results are supported by researchers who emphasize the importance of self-help when contacting by phone.
Does Providing Extended Relapse Prevention Bibliotherapy to Problem Gamblers Improve Outcome? David C. Hodgins, 2006	169 who had recently quit gambling were recruited *via* public ads and were followed for 6–12 months.	The results suggest that providing bibliotherapy to gamblers does not improve the result	Almost all participants reported having read the booklet provided by the researchers and in turn reported having used the procedures.
How Effective Is Bibliotherapy for Very Old Adults With Subthreshold Depression? A Randomized Controlled Trial Karlijn J. Joling, 2010	170 participants in retirement homes who were offered a self-help pamphlet adapted to the needs of the elderly population.	86% of participants completed the pamphlet, but no significant changes were found.	Bibliotherapy alone is not able to relieve the symptoms of depression in older populations
Bibliotherapy within a correctional setting Kenneth J. Kohutek, 1983	54 volunteers from the general and segregated sectors of a maximum security prison	There were no significant changes in the treatment but in the self-consciousness of the prisoners.	Bibliotherapy can have effects on the self-control and self-knowledge of the prisoners but has no distinction or greater effect than the therapeutic intervention.
Reading Group Rehabilitation for Patients with Psychosis: A Randomized Controlled Study Umberto Volpe, 2015	41 patients with psychosis were assigned to groups and evaluated at the beginning and end of the 6 months	Cognitive and psychosocial performance was improved after 6 months	The programs are an easy therapeutic implementation and are well regarded by patients.
A Bibliotherapy Approach to Relapse Prevention in Individuals with Panic Attacks Joseph Wright, PhD, 2000	participants who completed phase 2 of Febraro, Clum, Roodman, and Wright (1999) study. The materials were delivered by mail, without intervention or direct contact. Telephone calls were used to improve participation.	A mixed decrease but with a positive trend in the results for the improvement of participants' panic attacks.	Generally positive result, general report of fewer panic attacks
An evaluation of a collaborative bibliotherapy scheme delivered *via* a library service J. Mcdonald, 2012	157 participants of whom 114 gave their complete data. questionnaires and evaluations were offered.	Significant posttreatment improvement in men and women and in all groups.	The results support the theory that library-based bibliotherapy can be effective in treating mental disorders.
Manual-assisted cognitive-behavior therapy (MACT): a randomized controlled trial of a brief intervention with bibliotherapy in the treatment of recurrent deliberate self-harm K. Evans, 1999	34 patients between 16 and 50 years old who have suffered a parasuicidal attack in the last 12 months randomly assigned	32 patients are followed, of whom 10 carried out a suicidal act. The rates of depression and suicidal acts decreased.	This pilot study can be carried out effectively in cognitive behavioral therapy.
A Randomized Controlled Trial of Clinician-Supported Problem-Solving Bibliotherapy for Family Caregivers of People with First-Episode Psychosis Wai Tong Chien, 2016	116 caregivers assigned randomly and evaluated in periods of 1, 6, and 12 months.	Positive effects reflected in posttreatment evaluations at 12 months	The burden of care on the study subjects was alleviated and in turn family relationships improved
A controlled study of assisted bibliotherapy: an assisted self-help treatment for mild to moderate stress and anxiety Gary Winship 2010	a treatment group was compared with a controlled group, randomly assigned	The final results were carried out in only 43 participants due to the departure or decline of others. Significant improvements	Bibliotherapy is thought of as a more economical therapy alternative
Cognitive–Behavioral Bibliotherapy for Hypochondriasis: A Pilot Study Femke M. Buwalda, 2009	Participants were given a book with cognitive–behavioral activities	The results were positive, given the active participation.	Bibliotherapy can be an effective aid in the treatment of mental disorders.

Phenomenologically, each paper was read in accordance with real, sensible experiences described. Thus, the reading focused on real-life scenarios behind research subjects. Compared with a wait-list control group, individuals receiving relapse prevention (RP) exhibited significant reductions in the frequency of panic attacks, panic cognitions, anticipatory anxiety, avoidance, and depression. In addition, individuals in the RP group were more likely to attain a “clinically significant change” in status on both panic-free status and level of avoidance more frequently than individuals in the control group (23). For example, in an article focusing on panic attacks, subjects are written in as individuals, far beyond being mere topics of interest due to panic attacks.

Although the table may reflect the subjects as mere numbers or components in the study, for the purposes of the bioethical and value-based medicine research that is behind this, the subjects are taken as single phenomena, independent of numbers or statistics. The real element of value then is the final discussion and conclusions that each paper arrived at and how they reflected on the effectiveness and ineffectiveness—that bibliotherapy may have as treatment or how it could aid in treatment. Research on bibliotherapy yields benefits when teaching people about the value of literature and how it may impact their daily lives and their day-to-day practices. As was seen in the 13 articles reviewed for this study, bibliotherapy's results and effects vary across the board; however, the general consensus seems to be positive.

### A Road Map and Compass to Bibliotherapy as a Non-pharmaceutical Intervention

**Figure 4:**
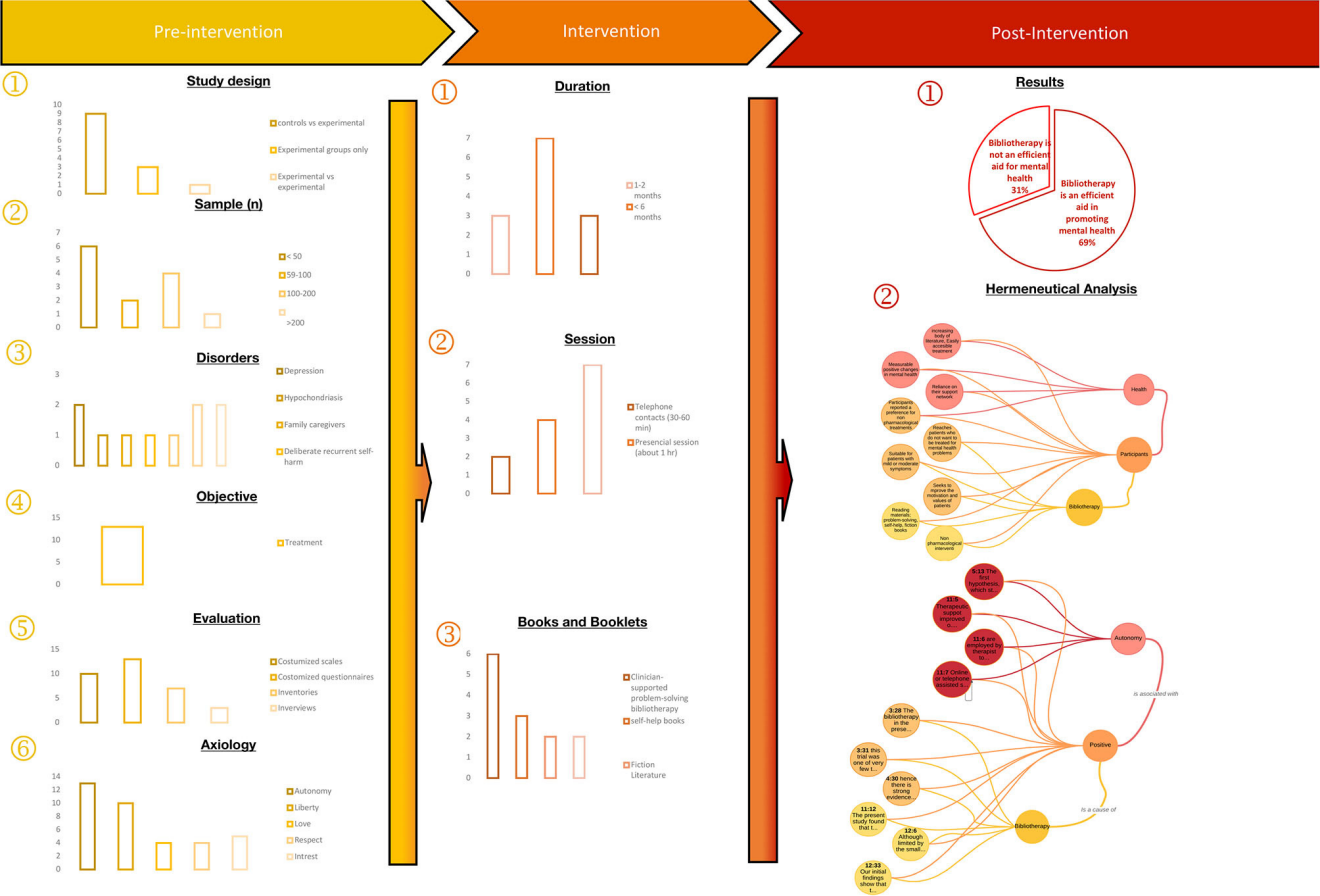
A road map to the systematic review. On the road map, we can appreciate the following: (1) That most of the studies on the last round of analysis were comparative experimental studies (9) that used a control group. (2) The studies most often had sample sizes smaller than 50 participants (6), but larger studies with less than 200 participants were also frequent. (3, 4) These studies tried to measure the efficacy of bibliotherapy mostly on patients with depression, anxiety disorders, and functional psychosis. (5) A variety of standardized tests, scales, and questionnaires were used along with interviews to measure the degree of change achieved through treatment. (6) Autonomy and liberty were the values most often related with positive results in these studies. (7) Bibliotherapy was offered in-person and through telephone sessions, although many of the studies did not pay enough attention to this aspect of the study. (8) A wide array of literature options was offered for these treatments, most frequently the clinician-supported problem-solving literature was used. (9) Nine out of the 13 studies reported positive results of bibliotherapy, which was considered a cost-efficient therapy suitable for mild to moderate disorders.

The results of prior studies illustrate some of the best practices that should be implemented in offering bibliotherapy (Figure 4). Several studies were designed to compare control and experimental groups, with relatively small groups (most of them with fewer than 50 participants). Most of the studies' length was between 3 and 6 months, and these were often the ones that had positive results. The aim of the studies was to treat disorders such as depression, stress, and anxiety, as well as functional psychosis, among others; it is an option that can be considered as far reaching for a large population, including HCWs, that has mental health problems during the COVID-19 pandemic (Figure 4).

It is important to note that there are still several features of bibliotherapy that need further research. For example, most of the studies found were not specific about the type of sessions in which the therapy was offered; the recurring types of sessions were in-person and through telephonic contact. The last of these options, as well as online contact, is especially important if we wish to offer mental healthcare during times in which health services may be needed to be reserved for those who are critically ill. Another feature of bibliotherapy, which needs further research, is the materials, books, and booklets used in this type of therapy. Out of 13 studies, six used problem-specific books to offer therapy; however, the structure of such materials is not clear and cannot easily be reproduced. Moreover, one should consider those who cannot easily access hospitals; thus, studies on the wider fiction literature that may help are needed.

## Discussion

The articles present themselves as sturdily scientific; however, their motivations are to aid patients who suffer from problems such as mental disorders, addiction, and imprisonment. The most valuable piece of information for the purposes of this study is the reports of the individual studies' subjects, as more patients reported feeling an improvement with the kind of bibliotherapy provided by the conductors of the investigations. Although none of the 13 articles directly discussed bibliotherapy in treating service workers, doctors, medical personnel, or even workers who suffer from burnout, some of the articles may be pointing in this direction, aiding us in our own implementation of bibliotherapy as an alternative intervention in mental healthcare. Studies that address topics such as anxiety, depression, caregivers, and stress-related issues may have a direct impact on the way we understand the approach to be taken for our own subjects.

This study specifically addresses the issue of values—or lack thereof—and their possible furtherance through literature while simultaneously encompassing the axiological works of several philosophers that shed light on this topic. The selection of literary works has been carefully curated to each reflect a distinct value, which will thereafter be applied in clinical practice through value-based medicine, which reflects and grapples with these absences in evidence-based medicine. One of the main objectives is to take the correct approach when trying to understand and, in a way, treat our subjects with the implementation of literature as a manner of therapy.

The methodological approach was based on these previous studies on the effectiveness of bibliotherapy. It is considered that, given the literature's effectiveness as a reflection medium, it could impact the attendees in their everyday practice (10). The most common therapeutic intervention used for bibliotherapy is CBT. CBT helps clients identify their distorted and depressogenic thinking and learn more realistic ways to frame their experiences by reading and conducting exercises that are completed at home, with minimal or no supervision from a therapist (10).

The results showed autonomy and justice linked with positive results in bibliotherapy often because this type of therapy could promote empowerment, decision-making, and problem-solving. Enhancement of clinical autonomy was also often reported where patients were prone to participate in their own treatment, reducing the caregiver's burden by improving compliance and reducing anxiety episodes. Though the role that reflection plays in reviewing the experience of others and how it strengthens self-control and decision-making is not clear, its effects are noticeable.

Bibliotherapy is a complementary resource to the clinical treatment of a disease. It is a strategy that helps patients, through literature, to cope with their situation by identifying with the experiences lived by the characters, and from reading, to develop their own tools to make better decisions about their health and exercise control over their lives and their illness. It is well-known that literature, as a reflection of human existence, leads those involved to reflect on themselves and their environment, and that, in addition to its esthetic character, it possesses the richness of confronting individuals with their emotions, values, feelings, and conflicts. It is also a way of helping individuals express, live, and solve these. It is an intrinsic character of literature to serve as therapy, catharsis, and cure for any conflict that disrupts our existence, and that is why human beings have always resorted to it (and, of course, also to the arts) in some way as the best medicine for life.

The process involves three phases: identification, catharsis, and insight (34). First, the reader creates a bond with the character with whom they identify most; then, this character encounters a conflict and resolves it; and finally, the reader, having experienced the conflict of the character through the text, reflects on personal circumstances and internalizes some behaviors represented in the book that will serve as tools to resolve their own conflicts. Nussbaum (35) for the same reason points out that “the novel's capacity to explore the length and breadth of a life, but the combination of this exploratory power with the presence of a character who will count as a high case of the human response to value, that creates the telling argument.”

The key to moral behavior not only implies theoretical understanding, but it must be connected with practice, unleashing a clear consciousness in the reader in such a way that unpacks their moral objectives, values, and hierarchy of values, creating moral abilities involved in reading and interpreting it (35, 36). Hence, patients can re-signify their own matters, being able to think about their lives and conflicts from a broader moral horizon.

In this study, we explored the heterogeneity of the outcome of bibliotherapy and its value network relationship. The results implied that the patient uses and develops several capacities in an indispensable way such as emotion, creativity, values, moral horizon, and imaginative capacity. This means that, as readers, we assume the challenge of unpacking our imperfections (such as physical, ethical, and axiological). Nussbaum (35) expresses it this way, “We notice the way we are inclined to miss things, to pass over things, to leave out certain interpretative possibilities while pursuing others.” In brief, to teach us “how we should live.” However, as Pellegrino [(37), p. 16] states: “She or he can enmesh us in the variegated particulars of an imagined life, but that cannot replace the hard work of normative ethics. In the end, the reader must choose whether to accept, reject, or modify his or her own way of life in light of the experience gained by the evocations of affect and thought in a work of fiction.”

Patients with psychosis improved in their clinical symptomatology and cognitive and psychosocial functioning after having attended a reading group program compared to patients who did not attend such structured activities. Patients who attended the group also reported that reading activity had a positive impact on group cooperation dynamics and that it was perceived as highly pleasant, useful, and interesting (22). Although not to be taken as a single means to treat a patient, it is an aid to other kinds of therapy such as CBT.

The scientific literature reports demonstrate certain benefits from bibliotherapy, maybe not surpassing those of other psychological treatments, but since one of the advantages of the treatment is that it can be widely available, bibliotherapy can and should be considered when developing public policies to help take care of the mental health of those affected by the COVID-19 pandemic, and for physicians, nurses, and other healthcare professionals to cope with the saturation of healthcare services during the COVID-19 pandemic. There is still plentiful details of the treatment and the phenomenon to discover and be systematically assessed to expand the benefits for health personnel and prevent diseases such as sleep disorders, anxiety, depression, and burnout, which greatly decrease the quality of life of communities and healthcare professionals. However, some thought must be given to the mechanisms of implementation of such therapies, where the most common instruments of bibliotherapy are books, which are currently difficult to share. In this sense, electronic books and materials would probably be a better option for implementation.

## Conclusion

After this systematic review, we respond to our main research question, building up the road map, and many conclusions regarding bibliotherapy can be drawn. First, when the methodology of a bibliotherapy treatment is conducted cautiously, positive effects can be seen, regardless of the diseases. It can be noted that bibliotherapy treatments promote values as supplementary profit. One of the main positive aspects of bibliotherapy is that it is a low-cost alternative that can reach those unable to access treatment during the COVID-19 pandemic; it is an integrative and multidisciplinary treatment that links psychology, medicine, humanities, and literature. Hence, this means that bibliotherapy could potentially be applied to a larger population and healthcare personnel and, when implemented in a structured way, could have a positive impact on enhancing mental health amid the COVID-19 pandemic.

## Data Availability Statement

The original contributions presented in the study are included in the article/Supplementary Material, further inquiries can be directed to the corresponding author/s.

## Author Contributions

MA-B, NA-B, PS, and AH-B conceived and designed the experiments. DM-F, IM-C, MA-B, AH-B, PS, and NA-B performed the systematic research and/or bioethical meta-analysis, and analyzed the data. DM-F, IM-C, MMA-B, SR, AH-B, PS, MA-M, and NA-B wrote the paper and contributed to helpful discussions. All authors contributed to the article and approved the submitted version.

## Conflict of Interest

The authors declare that the research was conducted in the absence of any commercial or financial relationships that could be construed as a potential conflict of interest.
