# Supplementary material for: Bibliotherapy as a Non-pharmaceutical Intervention to Enhance Mental Health in Response to the COVID-19 Pandemic: A Mixed-Methods Systematic Review and Bioethical Meta-Analysis
Source: Front Public Health. 2021 Mar 15;9:629872. doi: 10.3389/fpubh.2021.629872 (PMC8007779; doi:10.3389/fpubh.2021.629872)

SUPPLEMENTAL FILE

Bibliotherapy as a non-pharmaceutical intervention to enhance mental health in response to the COVID-19 pandemic: A mixed-methods systematic review and bioethical meta-analysis

Table 1S Codes and operational definition

CODE	Operational definition	CODE	Operational definition	CODE	Operational definition
**Honesty**	Is the complete disinterested presentation of information	**Charity**	Disinterested gift for the sake of another	**Only experimenta l**	When there was not a control group to compare to
**Veracity**	It is the capacity of expressing truthfully	**Purity**	Strictly acting under professional principles	**Participants**	When the study mentioned the characteristics and number of participants in the study
**Fidelity**	Attitudes that generate a confidence bond	**Beauty**	Capacity of recognize beauty standards	**Not relevant**	When a result from the study was not relevant

			according to a personal cosmovision		
**Justice**	Disposition to give each what is deserved	**Bibliotherapy**	When the study mentions bibliotherapy as an intervention.	**Positive**	When the result of the intervention was positive
**Autonomy**	This value refers to the ability of self-regulating one's own behavior	**Mental disorder**	When a targeted mental disorder was mentioned in the study	**Without differences**	When the intervention showed no differences between groups or situations
**Liberty**	The possibility of performing considering one´s own goals	**Sub-clinical problem**	When a sub-clinical problem was mentioned such as sadness, stress, happiness	**Descriptive**	When the result was only descriptive
**Equality**	Process of balance between two elements	**Survey**	When the study was about a survey and not a clinical trial		
**Respect**	A positive response and recognition of others' agency, identity and personhood	**Used instrument**	A instrument mentioned in the article		

**Toleran ce**	It is a positive response and a recognition of other´s behavior	**Sample**	When the article specified the sample, who participated
**Interest**	Disposition to generate a link with one another	**Control-experim ental**	If the study design compared a control group versus experimental group
**Love**	Capacity of affection that one can give to another person	**Experim ental-experim ental**	If the study design compared two different experimental groups
**Service Attitude**	Significant efforts made favoring the patients' care	**Treatme nt as usual-experim ental**	When an experimental intervention was compared to a usual intervention

Table 2S Families and codes of analysis

Families			
**Values**	**Themes**	**Methodological elements of the study**	**Conclusions and results**
**Honesty**	Bibliotherapy	Survey	Not relevant
**Veracity**	Mental disorder	Used instrument	Positive
**Fidelity**	Sub-clinic problem	Sample	Without differences
**Justice**		Control-experimental	Descriptive
**Autonomy**		Experimental-experimental	
**Liberty**		Treatment as usual-experimental	
**Equality Respect**		Only experimental	
**Tolerance**		Participants	
**Interest**			
**Love**			

Service
Attitude

Charity

Purity

Beauty

Fig 1S. It depicts the search strategy using the PIO model where P stands for Participants, I for Intervention, and O for Outcome. We defined the following keywords for participants: healthcare personnel, healthcare professional, healthcare manpower, physician, doctors, nurse, and social worker. The keywords for intervention were: bibliotherapy. And finally, the keywords for outcome were liberty, empowerment, tolerance, justice, benevolence, equity, respect, charity, beauty, autonomy, purity, ethical values, axiology, personal identity, and dignity. The figure describes the obtained results in each combination.

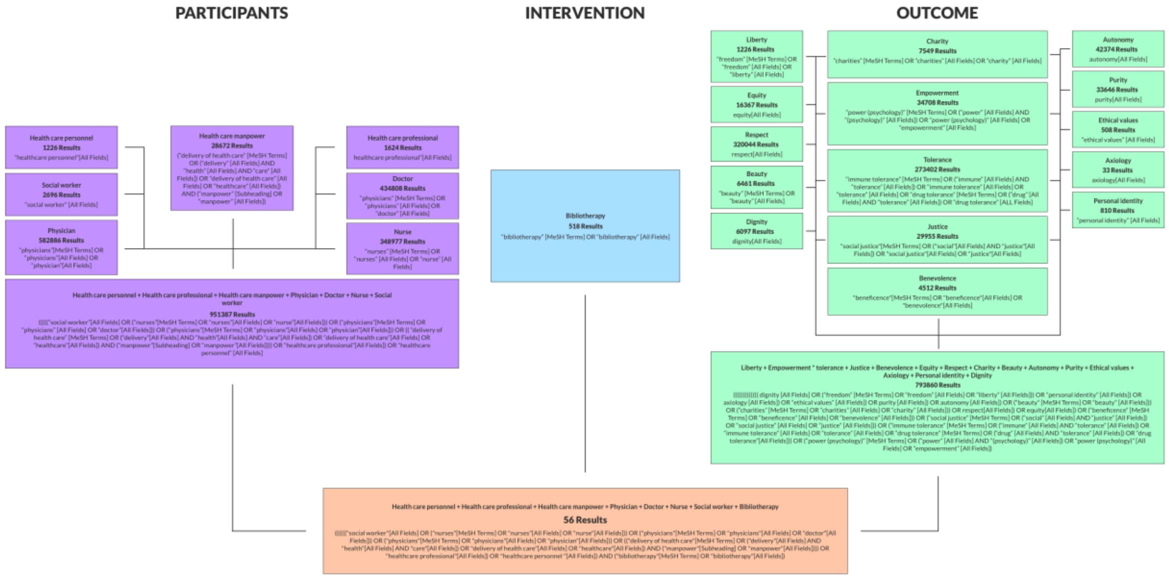

Supplement: Supplementary file 1 [file Data_Sheet_1.PDF]
